# Practical Dots

**Published:** 1886-01-20

**Authors:** T. L. Lallerstedt

**Affiliations:** Georgia


### PRACTICAL DOTS.

By T. L. LALLERSTEDT, M.D., OF GEORGIA.

*Gonorrhœa* is one disease in which the most remedies heretofore tried have given poorest general satisfaction. From some cause, about four years ago I commenced to use the fluid extract of rhus aromatica :

℞. Fluid ext. rhus aromatica......................℥ i,
Water......................................℥v. M.

Use as injection at least three or four times daily, the last time on retiring for the night—always evacuating the bladder before using the injection.

I always give special instructions to drink no liquors, avoid horseback riding and women.

In treating women with gonorrhœa, I either have them to put a sponge, that I give shape to, within the vagina, or, if no sponge is at hand, I use cotton batting; wet the sponge or batting well in the medicine, and replace about every six hours; the sponge or batting should be worn all the time. I have two sponges, and have the one not in use washed in boiling water.

I use the rhus aromatica for women as well as men, and, if an acute case, it often times cures in twenty-four hours. A few days ago I gave a man an 8-ounce bottle of the above prescription, who had gonorrhœa for eight months, and it cured him promptly. If I want to use a diuretic, I give fluid ext. gelsemium, 15 drops, three or four times a day, in a half glass of water.

*Irritation of Meatus urinarius.*—During the summer of 1883 I had a great deal of hard work to do, and long trips to make. I was attacked with acute cystitis, and tried almost everything I could read of, or any one would recommend, and found no relief. I was contemplating the abandonment of practice until I could get well. It had come to the point where I could not retain my urine more than a half hour at a time. The whole trouble seemed to be just at the meatus or end of the penis. It came into my mind, if I would insert morphine into the penis it might relieve me. I stopped at once and fixed me a small splinter—a smooth stick—with which I put $\frac{1}{4}$ grain of morphine sulph. into the meatus, and held it a few minutes, then went on to business. I was easy in a few minutes, and my urine gave me no trouble for nearly four hours. After urinating, the smarting was considerable. I then put in another dose of morphine, about $\frac{1}{8}$ of a grain, and I have been all right since.

*Chorea.*—It is not often that country doctors are called to treat "chorea," for it is a rare disease. I have seen two cases in the last seven years. The first was a young woman—a factory operative; she had been afflicted, more or less, for years. It was almost impossible to keep her on the bed. I first gave her a good dose of calomel and aloes, and then put her on the following prescription:

℞. Bromide of potassium....................grs. 960,
Tinct. of gelsemium..........................℥ i,
Water, q. s. to make.........................1 pint. M.

Dose, tablespoonful, every two hours, in glass two-thirds full of water, as long as the jerking continues; then every four hours, unless signs of jerking returns, in which case give as before.

About the second dose quieted her. She continued to take it every four hours until that pint was consumed ; then she continued the medicine in same amount, three times a day, until she took two more bottles—and she never had another attack. Once or twice she had some symptoms, when a good dose of the medicine relieved her promptly.

The second case was that of a boy about five years of age. He was first attacked with inflammation of the bowels, which gave way promptly to appropriate treatment. Just as he was getting able to sit up he was attacked with chorea ; but nothing I did for the first twenty-four hours was of much avail, for the reason that he would not swallow anything. On my second visit, I gave him enema of—

℞. Hydrate of chloral, }
Bromide of potassium, } aa......................grs. x,
Fluid ext. gelsemium..........................gtts. xv, in 2 ounces of starch.

In less than haif an hour he was perfectly quiet, and the effect of that dose did not pass off under twenty-four hours ; then I repeated same, and from about eighteen hours after giving the last enema I gave him 5 drops fluid ext. gelsemium, every hour and a half, in a little domestic wine. In a few days he had another attack of inflammation of the bowels, but it was soon relieved, and, as he gathered his strength, his diseases disappeared. I kept him on gelsemium for a good while—dose, 5 drops, three times a day. He is now well, and has been for several months.

*Headache* is a trouble that we country doctors are not often consulted about. I have, at least, two very potent remedies for headache, which I have always on hand : 1. Morphine sulph., hypodermically inserted as near the seat of pain as possible, from $\frac{1}{8}$ to $\frac{1}{2}$ grain. 2. Bromide of potass., ʒ i ; fluid ext. gelsemium, gtts. 40, in half glass or more of water, divided in two doses, half an hour apart. If I am at my office, and am aware what is the trouble, I always carry a bottle of bromo-caffein. We have never had as good results from any other agent. It is prompt in its action, and pleasant to take. Dose, from 1 to 2 teaspoonfuls in half a glass of water.

There is one remedy we use a good deal that we have not seen much written of, and hear little said about. Often the doctor is called to see cases of excessive vomiting from, to him, some unknown cause, and which may have been going on for hours. I find the patient cold, features pinched in appearance, and eyes gone back in head. I usually give small doses of calomel, and, if the patient is restless, a dose of morphine, and then, to stop the vomiting, I have applied to the whole length of the spine cloths wrung out of water as hot as can be borne. I have it folded several thicknesses; as one cools, apply another, and soon things will be better for your patient in every respect.

In *Metrorrhagia*—not at child-birth, but in other forms—I have found it speedy and safe to use the hot cloths. I have used on one occasion, in dysmenorrhœa, *cold* applications to spinal column with very best results. I relieved a patient of all pain, and brought on flow freely in this manner. I find this in Henry Hartshore's Principles and Practice of Medicine, page 97.

